# The effect of timing of incentive payments on response rates for cohort study telephone interviews in primary care setting with cost-minimization analysis, a randomized controlled trial

**DOI:** 10.1186/s12874-015-0073-3

**Published:** 2015-10-06

**Authors:** Weng-Yee Chin, Edmond PH Choi, Cindy LK Lam

**Affiliations:** Department of Family Medicine and Primary Care, The University of Hong Kong, 3/F., 161 Main Street, Ap Lei Chau Clinic, Ap Lei Chau, Hong Kong; School of Nursing, The University of Hong Kong, 4/F, William M.W. Mong Block, 21 Sassoon Road, Pokfulam, Hong Kong

**Keywords:** Incentive, Telephone interview, Response rate, Longitudinal study, Cost minimization, Chinese population

## Abstract

**Background:**

The effect of timing of incentive payments on the response rate of telephone surveys is unknown. This study examined whether up-front or delayed incentive payments were associated with higher response rates for participation in a telephone interview administered longitudinal cohort study amongst primary care patients with lower urinary tract symptoms, and to compare the costs between the two timing methods.

**Methods:**

This study was conducted as part of a naturalistic observation study on the health-related quality of life and health outcomes of Chinese primary care patients with lower urinary tract symptoms. The incentive payment was in the form of a supermarket gift voucher to the value of HD$50 (US$6.50) and could be used in lieu of cash at a major supermarket chain.720 subjects with lower urinary tract symptoms were randomly assigned into two groups. One group was offered an incentive of supermarket cash voucher at time of recruitment (‘up-front’ payment). The other group was told that the voucher would be sent to them after the complete of their 1-year follow-up telephone interview (‘delayed’ payment). Primary outcomes were the baseline and 1-year follow-up telephone survey response rates.

**Results:**

There was no statistical difference in response rates at baseline (*p*-value = 0.938) or at the 1-year follow-up (*p*-value = 0.751) between groups. Cost per completed subject interviews for the up-front payment method was USD16.64, whilst cost for the delayed payment was USD 13.85.

**Conclusions:**

It appears the timing of incentive payments does not affect response rates for telephone interview surveys conducted on primary care patients in Hong Kong at baseline or at 1-year follow-up. Delayed incentive payments can reduce the overall cost per successful case.

**Trial registration:**

ClinicalTrials.gov Identifier: NCT02307929 Registered 28 August 2013

## Background

Patients are frequently approached for surveys because they are of considerable research interest. Using surveys to collect patient self-reported information is very common in health services research, such as epidemiological studies [1], clinical trials [2] and patient satisfaction studies [3].

Telephone surveys are commonly used for collecting data in longitudinal studies of patient populations. There are significant advantages to using telephone interviews to administer surveys: it reduces the amount of time a patient needs to spend at the doctor’s clinic; it removes the need for subjects or interviewers to travel; and interviews can be conducted outside of business hours when respondents are more available. Overall, it is more convenient for the subject and can potentially help to enhance response rates for longitudinal studies that require patient self-reported information. Telephone interviews are often preferable to postal or online surveys as the interviewer can help to ensure the validity and reliability of responses by reducing missing data and providing item clarification to the respondents [4]. This is particularly important in patient population settings, where subjects are often elderly who may have a visual impairment or in populations with poor literacy. Furthermore, an essential component for most survey studies is an adequate response rate to reduce non-response or self-selection bias [5, 6].

Many studies have found that incentive payments can enhance the response rates for postal and telephone surveys [7, 8]. However, findings about the effect of timing of incentive payments on response rates have been conflicting. A meta-analysis on the effects of incentive payment in cross-sectional postal surveys found that compared to delayed incentive payments, up-front incentive payments can increase the response rates of 11.9 percentage [7]. A Cochrane review which included 24 trials evaluating the timing of incentive payments for postal questionnaires found that the odds of receiving a response increased by more than a half for up-front payments, compared with delayed payments (odd ratio: 1.61; 95 % confidence interval 1.36 to 1.89) [9]. Previous studies have adopted the social exchange theory to explain up-front incentive payments can increase response rates [6, 10, 11]. It was suggested that “the norm of reciprocity” plays a role in compliance to requests [12, 13]. Requesters can trigger the reciprocity by offering an unsolicited favor. Recipients of a gift or incentive are more likely to agree to a subsequent request than someone who did not receive any favors [12]. There are two explanations. First, people return favors because they are concerned what the other people think of them they do not want to be perceived as ‘free riders’ [14]. Second, people feel good about themselves when they return a favor [12]. Up-front incentive payments can thus initiate an exchange that influences people to feel obligated to complete the survey. Accepting an up-front incentive payment without answering the subsequent telephone survey might make people feel guilty, according to the norm of reciprocity in the social exchange theory [6, 10, 11, 15]. On the contrary, delayed incentive payment imposes little social obligation on people. The respondent with a delayed payment option is the one who holds controls over the initiation of an exchange. Before a respondent answers a survey, no obligation exists on either requester or respondent [6, 11].

On the contrary, the Cochrane review found that the timing of incentive payments had no effect for electronic questionnaire response rates (odd ratio: 1.08; 95 % confidence interval 0.77 to 1.50) [9]. Furthermore, another meta-analysis found that there was no difference in response rates between up-front and delayed incentive payments for interviewer-mediated surveys, including face-to-face and telephone interviews [8].

However, there are still some knowledge gaps. First, in previous studies, the mode of questionnaire administration has been mainly by self-administration [9]. The effect of timing of incentive payments on the response rate of interviewer-administered questionnaire, especially for telephone surveys, is still unknown. Second, previous studies have been predominantly conducted in non-patient populations, such as in the general population, students, technicians, corporate settings and medical personnel [7, 9]. Third, most studies to date have been cross-sectional. Little is known about the effect of timing of incentive payments on the response rates to longitudinal telephone surveys in primary care patient settings.

In order to understand the impact of timing of incentive payments on response rates, we conducted supplementary and parallel work on our naturalistic observation study on Chinese primary care patients with lower urinary tract symptoms [16]. The aim and objectives, and the study methodology of the main study have been reported elsewhere [16–19]. One of the advantages to conducting the investigation in a primary care population was that we could approach the notion of general population in primary care while avoiding some of the complexities of having to sample the general population. Second, telephone survey is preferable in studies in primary care where the doctor’s consultation schedules are tight with many patients attending the doctor in a short period of time. Compared with face-to face interviews, telephone surveys can save the patient’s time at the clinic; enhance the number of patients recruited; and is less disruptive on the delivery of medical services.

The present study aimed to examine whether up-front or delayed incentive payment was associated with a higher response rate for telephone interviews in a longitudinal cohort study on primary care patients, and to compare the cost between the two incentive payment methods.

## Methods

### Subjects

This single-blinded randomized controlled trial with cost-minimization analysis was conducted as part of a naturalistic observational study on Chinese primary care patients with lower urinary tract symptoms (LUTS) [16]. There were two sampling frames for the study.

#### Sample A (Continence care clinic)

All new patients referred for services at a nurse-led continence care clinic at one of four government-funded General Out-Patient clinics (GOPC) across Hong Kong were identified from the appointment list and screened for LUTS. All eligible subjects were invited to participate in the study.

#### Sample B (GOPC waiting room)

Waiting room patients waiting to consult a doctor at one of another four GOPC locations were consecutively screened for LUTS. All eligible subjects were invited to participate in the study.

For both sampling frames, a modified International Consultation on Incontinence Questionnaire-Urinary Incontinence-Short Form (ICIQ-UI SF) questionnaire was used to screen for patient eligibility [20]. Patients with scores ≥ 3 were considered to have LUTS. Patients were excluded if they were aged < 18 years, could not understand Cantonese, refused to participate, or were too ill to give consent.

Eligible patients were identified and consecutively approached by trained field workers to invite them to take part in the longitudinal telephone survey. The aims, procedures and nature of the naturalistic observation study were explained. Patients were also informed about the timing of incentive payments (either up-front or delayed incentive payment), according to the randomization list. Subjects were blinded to the alternate payment method. In other words, patients who were assigned to receive up-front an incentive payment did not know there was a delayed incentive payment group and vice versa. Subjects who consented were asked to provide their contact details for telephone surveys that were administered by blinded interviewers who were not aware of the incentive payment method allocation.

All patients were informed at the time of consent that both interviews (baseline and follow-up) would take approximately 15 min to complete.

### Randomization

Subjects were assigned to either the up-front incentive payment group or delayed incentive payment group by simple randomization on a 1:1 basis with random numbers generated by a computer. A statistician who was not involved in subject recruitment or patient interviews undertook the randomization.

### Interventions

The incentive payment was in the form of a supermarket gift voucher to the value of HD$50 (US$6.50) and could be used in lieu of cash at a major supermarket chain.

#### Up-front payment group

Each subject was handed a supermarket gift voucher valued at HKD$ 50 (US$6.50) at time of recruitment immediately following consent to participate. They were required to sign a receipt confirming they had received the voucher. There was no extra administrative work for the incentive payment afterwards.

#### Delayed payment group

We collected the mailing address of each subject at the time of enrollment. Each subject was posted a supermarket gift voucher valued at HKD$50 (US$6.50) with a self-addressed envelope for the return of a signed receipt slip. This was sent after the completion of their 1-year follow-up telephone interview. Prior to posting the gift vouchers, each subject was phoned to confirm the address and reminded to return a signed receipt by post to confirm that they had received payment.

### Data collection

Subjects were interviewed by telephone two weeks after recruitment (baseline) and again at 1-year after the baseline interview by trained interviewers who were blinded to the randomization. Trained interviewers administered all surveys with a computer-aided telephone interviewing system between 10:00 am and 10:30 pm on weekdays. A maximum of five attempts were made for unanswered calls. Subjects answered a structured questionnaire on the severity of LUTS, health-related quality of life, mental health, service utilization, co-morbidities and socio-demographics. There were 77 items in the baseline interview and 51 items in the 1-year follow-up interview.

### Outcomes

Primary outcome: baseline and 1-year follow-up telephone survey response rates.

Secondary outcome: the cost per subject who had completed both baseline and 1-year surveys.

### Statistical analysis

Baseline socio-demographic and clinical characteristics between both groups were compared by chi-square test for categorical variables, and by independent t-test for continuous variables. Chi-square test was used to compare the consent and response rates at baseline and 1-year follow-up telephone surveys between groups. The cost minimization analysis included all relevant direct costs including the cost of the supermarket voucher, staff costs for the manpower administering the baseline and 1-year telephone interviews, staff costs for the administrative work related to the mail out of the incentive payments as well as the cost of consumables such as postage and stationery. The cost per completed survey at 1-year for both groups was calculated.

A meta-analysis study on mail survey response rates found an overall response rate of 53.0 % for up-front incentive payment and 41.1 % for delayed incentive payments [7]. Using this, to achieve 90 % power to detect a difference between the group proportions by Chi-square test with a 0.05 significance level, an estimated sample size of 360 subjects was needed in each group.

### Ethics approval

The study protocol of the present study was approved by the institutional review boards: the University of Hong Kong/Hospital Authority Hong Kong West Cluster, Hong Kong East Cluster Kowloon West Cluster, and Kowloon East Cluster. Written informed consent for participation in the study was obtained from participants.

## Results

**Fig. 1:**
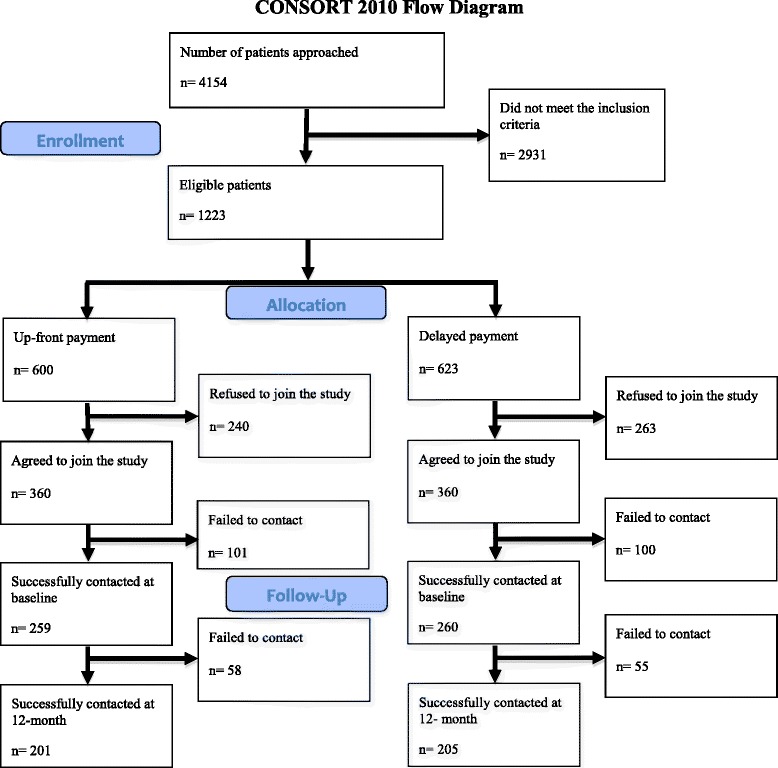
Subject recruitment and follow-up flow chart

The subject recruitment period was from March 2013- July 2013. The follow-up period was from March 2014- July 2014. Figure 1 shows the subject recruitment and follow-up flow chart. A total of 720 patients consented to participate with a response rate of 58.8 % of all eligible patients approached. 720 subjects were randomly assigned to either the up-front payment group or delayed payment group (360 subjects in each group).

Table 1 shows the basic socio-demographic between two groups. There was no statistical difference in baseline characteristics between two groups.

**Table 1:** Socio-demographic and baseline characteristics

	Up-front payment group	Delayed payment group
Mean age in years (SD)	63.9 (11.2)	63.9 (11.5)
Genders (n)		
Male (%)	105 (40.5 %)	124 (47.7 %)
Female (%)	154 (59.5 %)	136 (52.3 %)
Employment status (n)		
Working (%)	84 (32.4 %)	95 (36.5)
Not working (%)	175 (67.6 %)	165 (36.5)
Smoking status (n)		
Smoker (%)	53 (21.3 %)	59 (24.2 %)
Nonsmoker (%)	196 (78.7 %)	185 (75.8)
Drinking status (n)		
Drinker (%)	108 (43.5)	128 (52.2 %)
Nondrinker (%)	140 (56.5 %)	117 (47.8 %)

Chi-squared test showed *P* > 0.05 for all categorical variables and t-test showed *P* > 0.05 for all continuous variables

Table 2 shows the consent rates and response rates at baseline and at the 1-year follow-up between groups. In the up-front payment group, 259 subjects (71.9 %) completed the baseline telephone survey whilst 201 subjects (77.6 %) completed the 1-year telephone survey. In the delayed payment group, 260 subjects (72.2 %) completed the baseline telephone survey whilst 205 subjects (78.8 %) completed the 1-year telephone survey. The timing of incentive payment had no effect on whether patients agreed to join the study or not (*P* value = 0.433). There was no statistical difference in response rates at baseline (*P* value = 0.938) and at 1-year follow-up (*P* value = 0.751) interviews between two groups.

**Table 2:** Response rates between incentive payment groups

	Up-front payment group	Delayed payment group	Chi-square test
Patients contacted (n)	600	623	
Patients who refused to join the study n (%)	240 (40 %)	263 (42 %)	*p* = 0.433
Patients who agreed to join the study n (%)	360 (60 %)	360 (58 %)	
Baseline interview n (%)			
Successfully interviewed	259 (71.9 %)	260 (72.2 %)	*p* = 0.938
Failed to contact	101 (28.1 %)	100 (27.8 %)	
12-Month follow-up interview n (%)			
Successfully interviewed	201 (77.6 %)	205 (78.8 %)	*p* = 0.751
Failed to contact	58 (22.4 %)	55 (21.2 %)	

Table 3 shows the cost per subject who completed the 1-year follow-up interview. The cost per completed subject interview for the group with up-front incentive payment was USD 16.64, which was higher than the cost per completed subject for the group with delayed incentive payment of USD 13.85.

**Table 3:** Cost per subject who completed baseline and follow-up interviews

		Up-front payment		Delayed payment	
Resource component	Unit cost (USD)	n	n X unit cost (USD)	n	n X unit cost (USD)
Incentive					
Coupon	6.50	360	2340	205	1332.50
Staff					
Baseline interview^a^	2.18	259	565.66	260	567.84
12-month interview	2.18	201	438.98	205	447.72
Administrative work for payment at 12-month^b^	1.82	0	0.00	205	373.10
Stationary					
Letter to patient at 12-month	0.01	0	0.00	205	1.68
Postage	0.44	0	0.00	205	90.61
Two envelopes	0.12	0	0.00	205	25.05
Total cost (USD)			3344.64		2838.50
Number of subjects successfully contacted at 12 months			201		205
Cost (USD)/successful subject at 12 months			16.64		13.85

^a^Calculation was based on 12 mins/subject to process each incentive payment and an administrative staff cost of 10.9 USD

^b^Calculation was based on 10 mins/subject to process each incentive payment and an administrative staff cost of 10.9 USD

## Discussion

To our knowledge, this was the first randomized experimental study to examine the effect of the timing of incentive payments on response rates and cost-minimization analysis in a patient population. We found the timing of incentive payments (up-front vs. delayed incentive payments) had no effect on consent rates and completion rates for the telephone survey at baseline and at 1-year. The findings were similar to those of previous studies [21, 22]. There was no difference in response rates of a mailed survey between up-front incentive and delayed payment in prostate cancer survivors [21] and breast cancer survivors [22]. However, our findings were contrary to most of the existing evidence [7, 9, 11]. The finding of the Cochrane review and Church’s meta-analysis suggested that up-front incentive payment could significantly increase the response rate of postal surveys [7, 9]. In two studies examining physician’s response to postal surveys, when compared with delayed incentive payments, up-front payments were associated with higher response rates such as (78 % vs. 66 %) [23] and (71 % vs. 54 %) [11]. It should be noted that these studies used postal rather than telephone surveys in non-patient populations and as a result, such findings may not be transferable to telephone-interviewer administered surveys of patient populations. Furthermore, it appears that the explanations derived from the social exchange theory cannot explain the result of the present study.

One possible reason for our findings is because we studied a public-sector patient population. Traditionally, according to the sick role theory [24], patients have an obligation to cooperate with the medical professional, such as adhering to medical regimen and research participation. A qualitative study about the belief of African Americans toward medical research participation found that some older participants thought that medical research participation was beneficial to their family and society [25]. It is possible that patients completed the survey for the sake of obligation or altruism and the timing of incentive payment was not their primary concern. Furthermore, similar to the studies on cancer survivors [21, 22], many of our primary care patients had chronic diseases, and may have been interested in surveys that target issues related to their specific health condition. Therefore, we could not find any difference in consent and response rates between two groups. Further qualitative study to explore the attitudes and belief of patients toward incentive payment in medical research participation should be conducted.

The mode of survey administration using telephone interview rather than a postal survey may also have contributed to our results. Subjects need more motivation to take part in postal surveys due to the effort required to fill out the questionnaire and post it back. Hence, for those with delayed payment in postal survey, a higher degree of effort is required for the reward. Using a telephone survey, subjects play a more passive role, making it easier to fulfill the requirements to gain the reward. Respondents of our telephone interviews only needed to make themselves available to answer questions, and with the widespread use of mobile telephones, were able to respond even whilst participating in other activities. Our respondents therefore had fewer barriers in completing the telephone survey. Furthermore, it is also possible that respondents may have felt guilty in rejecting our interviewers since there was an actual person on the other end of the phone.

In the present study, we found that delaying the incentive payment helped to minimize the cost per successful case because only those who completed the 1-year telephone interview were rewarded. Our result was contrary to a previous study which found that up-front payment was less expensive than delayed payment [26]. However, the processing of delayed payments (including postage, delivery and confirmation of its receipt) caused a significant increase in administrative burden. For the delayed payment group, we needed to send the incentive payment to patients by post and for respondents to return a signed receipt to confirm that they had received payment. It incurred double postage costs as well as duplication of other miscellaneous expenses such as stationary and printing costs. Most importantly, it was very labor-intensive work in terms of research assistant staff time. On the contrary, by using up-front incentive payments it was possible to save both staff and postage costs.

### Recommendation

We found no difference in response rates between two payment methods. Up-front payment is preferable because it can save on the additional staff workload in administering the delayed incentive payments such as confirming the address of recipients, issuing letters and collecting signed receipts. Importantly, a loss of mail is problematic. However, if a study’s budget is tightly limited, incentive payments should be offered at the completion of the follow-up survey because it can minimize the cost per successful subject.

### Limitation

There are several limitations to this study. All subjects in this study were recruited from public (Government subsidized) primary care settings. The findings might not be generalizable to other settings or non-patient population. Besides, only subjects with LUTS were recruited into this study that was studying the impact of their condition on self-reported health related quality of life. Our subjects were likely to be more motivated to respond, as the topic was highly relevant to their own health. Further studies should be conducted in general patient population in order to confirm our findings. This study used a telephone survey and our findings may not be generalizable to other data collection methods such as online surveys. The cost-minimization analysis is based on the rates for University of Hong Kong’s research staff and postage costs, which may not be applicable to other settings.

## Conclusions

In our study setting of Hong Kong primary care patients, there was no association between the timing of incentive payment and response rates at baseline or 1-year follow-up telephone interview administered surveys. Delayed incentive payment can minimize the cost per successful case but produces more administrative burden.

## Abbreviations

LUTS: Lower urinary tract symptoms
GOPC: General out-patient clinics
ICIQ-UI SF: International consultation on incontinence questionnaire-urinary incontinence-short form
HKD: Hong Kong dollar
USD: United States dollar
